# Genomic Prediction for Grain Yield and Yield-Related Traits in Chinese Winter Wheat

**DOI:** 10.3390/ijms21041342

**Published:** 2020-02-17

**Authors:** Mohsin Ali, Yong Zhang, Awais Rasheed, Jiankang Wang, Luyan Zhang

**Affiliations:** 1 National Key Facility for Crop Gene Resources and Genetic Improvement, and Institute of Crop Sciences, Chinese Academy of Agricultural Sciences (CAAS), Beijing 100081, China; mali1990@yahoo.com (M.A.); zhangyong05@caas.cn (Y.Z.); wangjiankang@caas.cn (J.W.); 2 International Maize and Wheat Improvement Center (CIMMYT) China Office, c/o CAAS, 12 Zhongguancun South Street, Beijing 100081, China; arasheed@qau.edu.pk; 3 Department of Plant Sciences, Quaid-i-Azam University, Islamabad 45320, Pakistan

**Keywords:** wheat, genomic selection, missing data, minor allele frequency

## Abstract

Genomic selection (GS) is a strategy to predict the genetic merits of individuals using genome-wide markers. However, GS prediction accuracy is affected by many factors, including missing rate and minor allele frequency (MAF) of genotypic data, GS models, trait features, etc. In this study, we used one wheat population to investigate prediction accuracies of various GS models on yield and yield-related traits from various quality control (QC) scenarios, missing genotype imputation, and genome-wide association studies (GWAS)-derived markers. Missing rate and MAF of single nucleotide polymorphism (SNP) markers were two major factors in QC. Five missing rate levels (0%, 20%, 40%, 60%, and 80%) and three MAF levels (0%, 5%, and 10%) were considered and the five-fold cross validation was used to estimate the prediction accuracy. The results indicated that a moderate missing rate level (20% to 40%) and MAF (5%) threshold provided better prediction accuracy. Under this QC scenario, prediction accuracies were further calculated for imputed and GWAS-derived markers. It was observed that the accuracies of the six traits were related to their heritability and genetic architecture, as well as the GS prediction model. Moore–Penrose generalized inverse (GenInv), ridge regression (RidgeReg), and random forest (RForest) resulted in higher prediction accuracies than other GS models across traits. Imputation of missing genotypic data had marginal effect on prediction accuracy, while GWAS-derived markers improved the prediction accuracy in most cases. These results demonstrate that QC on missing rate and MAF had positive impact on the predictability of GS models. We failed to identify one single combination of QC scenarios that could outperform the others for all traits and GS models. However, the balance between marker number and marker quality is important for the deployment of GS in wheat breeding. GWAS is able to select markers which are mostly related to traits, and therefore can be used to improve the prediction accuracy of GS.

## 1. Introduction

Wheat (*Triticum aestivum* L.) is one of the major cultivated crops that is growing on approximately 200 million hectares worldwide and delivers one fifth of the total caloric demands of the global population [1]. The increasing population and climate fluctuations impose new breeding challenges and require wheat breeders to use more efficient selection methods to develop high-yield cultivars with multiple resistances and wide adaptations [2]. Improvement of grain yield is still a considerable challenge to wheat breeding and production. Hence, modern wheat breeding approaches, such as the combination of accurate or suitable experimental designs, multiyear and multilocation trials, the application of concepts of quantitative and population genetics, and the integration of various disciplines such as computer science, statistics, and mathematics have been utilized widely in the last decade [3].

Recent advancements in high-throughput sequencing platforms have generated genome-wide dense molecular markers for genetic analysis in wheat [4]. Genomic selection (GS) is a special type of marker-assisted selection that incorporates genome-wide dense markers, as proposed by Meuwissen et al. [5]. GS could be a powerful tool in crop breeding to improve the prediction and selection accuracy for quantitative traits [6]. GS utilizes one or more training populations (TP) that have been genotyped and phenotyped to calibrate or train a statistical model. Then, the trained model is used to predict genomic estimated breeding values (GEBVs) in a validating population (VP), which is only genotyped. Superior parents for the next breeding cycle are selected based on the GEBV and consequently reduce the generation interval. Generally, the number of markers used for training the statistical model is far larger than the number of observations. Whole-genome regression methods based on ordinary least squares cannot estimate all marker effects simultaneously due to insufficient degrees of freedom. To address this issue, various classical statistical, Bayesian, and machine learning methods have been proposed for predicting the genetic merits of individuals [7]. These methods differ from each other mainly by a range of assumptions in the estimation of breeding values and variances in quantitative traits and computational complexity [2,7]. Among the parametric models, ridge regression (RidgeReg), ridge regression best linear unbiased predictions (RRBLUP), and genomic-BLUP (GBLUP) assume the normal distribution of marker effects with equal variance [7]. Least absolute shrinkage and selection operator (LASSO), Bayes A, and weighted Bayesian shrinkage regression or nonlinear regression assume the prior distribution of marker effects with a high probability and moderate to large effects, while Bayes B and Bayes Cπ assume some marker effects to be zero [7]. Nonparametric or semiparametric models, such as random forest (RForest), reproducing kernel Hilbert space (RKHS), and neural network approaches, have also been applied in GS [6,8,9]. Nonparametric models, such as RForest and RKHS, are capable of capturing non-additive effects and complex and nonexplicit interactions [2,9]. Previous efforts to compare the predictive ability of various GS models in wheat showed the good performances of RF and RKHS for traits of interest, but no single GS model outperformed the other models in all cases [9,10].

The efficiency of GS is always expressed by prediction accuracy, i.e., the correlation coefficient between observed phenotypic values and predicted GEBVs in VP. Previous studies have indicated that many factors are interrelated in a comprehensive manner [7,11], such as the genetic architecture of traits [11,12], heritability, population structure [13], type of statistical models, i.e., parametric and nonparametric models [9], cross-validation strategies [12], training population size and composition [12,13], marker density [6,13], and linkage disequilibrium (LD) between markers and QTL. Recent studies in animal and plant breeding have demonstrated that quality control (QC) on markers can improve the prediction accuracy of GS [14,15]. However, studies on the effect of missing rate and minor allele frequency (MAF) QC on the prediction accuracy of yield and yield-related traits are limited in wheat.

GS holds potential for the genetic improvement of qualitative and quantitative traits and has been widely used in wheat breeding to predict various traits, such as grain yield [12], test weight, heading time [10,12], disease resistance [16], end-use quality [17], iron and zinc contents [18], and physiological traits [19]. In addition, some studies have described the practical applications of GS in wheat breeding, such as cultivar development [20], cross prediction [21], and heterosis [22]. In this study, a wheat training population was developed from 166 elite wheat cultivars collected mainly from China. More than 80% of the cultivars (144) were collected from the Yellow and Huai River valley of China, which is one of the most important agricultural regions of wheat production in China and has an area of approximately 15 million hectares [23]. The main objectives of this study were (1) to evaluate the performance of seven GS models in predicting yield and yield-related traits in this wheat population, (2) to assess the effects of missing rate and MAF QC on the prediction accuracy of GS models, (3) to evaluate the effect of genotype imputation and genome-wide association studies (GWAS)-derived markers on prediction accuracy of GS.

## 2. Results

### 2.1. Phenotypic Evaluation

The descriptive statistics of grain yield (GY) and yield-related traits, i.e., spike number per square meter (SN), thousand-kernel weight (TKW), spike length (SL), heading days (HD), and plant height (PH) of the 166 wheat accessions in different environments (locations in cropping seasons) are shown in Table S1. The average values in each environment ranged from 6320.25 to 9318.19 kg per hectare (kg·ha^−1^) for GY, 534 to 693 for SN, 39.38 to 49.82 g for TKW, 8.83 to 9.64 cm for SL, 184 to 199 days for HD, and 77.60 to 91.52 cm for PH (Table S1). Overall, the averages of BLUE values for GY, SN, TKW, SL, HD, and PH across all environments were 7268.81 kg·ha^−1^, 605, 43.17 g, 9.15 cm, 187 days, and 83.36 cm, respectively (Table S1). High heritability was observed for all traits in all environments and ranged from 0.70 (for SL) to 1 (for HD). The difference in heritability among traits reflected the contribution of the environment to variations across locations and years (Table S1).

**Figure 1:**
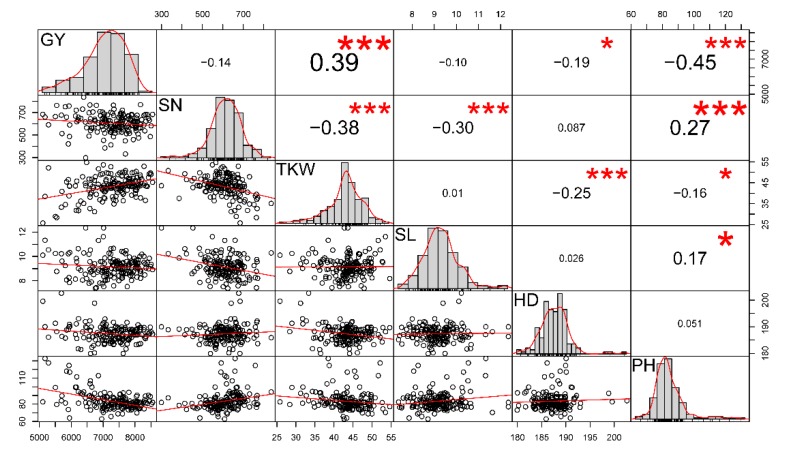
Pearson’s correlation matrix among yield and yield-related traits based on their best linear unbiased estimates (BLUE). The upper corner represents the correlation coefficient, with the significance level indicated by asterisks. Three symbols (“*” and “***”) correspond to three *p*-values (0.05 and 0.001, respectively). The lower corner contains bivariate scatter plots with fitted lines. The diagonally arranged plots show the phenotypic distribution of traits based on BLUE values. GY, indicates grain yield; SN, spike number per square meter; TKW, thousand-kernel weight; SL, spike length; HD, heading days; PH, plant height.

The Pearson’s correlation coefficient (*r*) between traits ranged from −0.45 to 0.39 (Figure 1). Under the significance level of 0.001, GY had the highest positive correlation with TKW (*r* = 0.39) and lowest negative correlation with PH (*r* = −0.45, Figure 1). SN was negatively correlated with TKW (*r* = −0.38) but positively correlated with PH (*r* = 0.27). TKW was negatively correlated with HD (*r* = −0.25).

From ANOVA across environments, the genotype, block, environment, and genotype-by-environment interaction effects were all significant at a level of 0.001. For TKW, SL, and PH, the variance of environment and genotype-by-environment interaction was lower than the genotypic variance (Table 1). Environmental variance was the highest for HD, and the variance of the genotype-by-environment interaction was the largest for GY. Plot-level heritability was high for PH (0.85), HD (0.81), and TKW (0.77) but was relatively low for GY (0.42) (Table 1).

### 2.2. Marker Coverage, Genetic Diversity, and Linkage Disequilibrium Analysis

A total of 11,997 SNPs from 90 K of genotypic data were chosen to create two QC scenarios that were used for genomic prediction. In the first scenario, five subsets of markers were generated by removing markers with missing rate values above or equal to different thresholds (0%, 20%, 40%, 60%, and 80%). In the second scenario, three subsets of markers were generated by removing markers with MAF levels under or equal to different thresholds (0%, 5%, and 10%) for each missing level. The number of SNPs decreased significantly after the application of missing rate and MAF QC (Table 2). The distribution of these markers on the 21 wheat chromosomes is shown in Table S2. Markers were unevenly distributed along chromosomes. Generally, for the MAF level of 0%, the B genome had more markers than the A genome, and the A genome had more markers than the D genome; for the other MAF levels, the A genome had the most markers, and the D genome had the least markers (Table S2). The estimated polymorphic information content (PIC) values ranged from 0.005 to 0.702 across wheat accessions, with an average value of 0.13, whereas the genetic diversity (GD) ranged from 0.006 to 0.749, with a mean value of 0.149 (Figure S1).

In total, 9851 SNPs with missing rate levels <40% were used to evaluate LD decay across the whole genome. The average *r*^2^ was 0.065 in the whole genome, and the average LD decay distances for 10, 100, and 10,000 Mb were estimated to be 0.38, 0.28, and 0.14, respectively. The scatter plot between *r*^2^ and physical distance (Mb) showed that LD decreased with increasing physical distance (Figure S2).

### 2.3. Prediction Accuracy of Different GS Models under Different Missing Rate and MAF Levels

**Figure 2:**
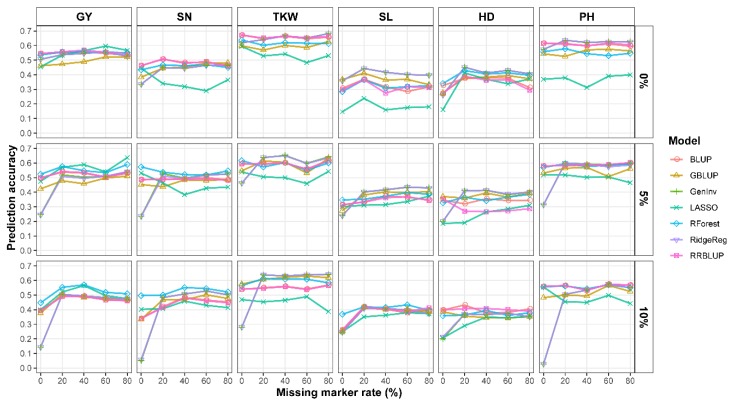
Genomic prediction accuracy of seven genomic selection (GS) models for yield and yield-related traits with five missing rates (columns) and three minor allele frequencies (MAFs) (rows). 0% MAF, represents markers with MAF greater than 0; GY, indicates grain yield; SN, spike number per square meter; TKW, thousand-kernel weight; SL, spike length; HD, heading days; PH, plant height.

Seven GS models were evaluated in this study. The prediction accuracies of the GS models ranged from 0.026 (PH) to 0.682 (TKW) and varied significantly among the six traits, five missing rate levels, and three MAF levels (Figure 2). Overall, QC for the missing rate and MAF improved the prediction accuracy, irrespective of traits and GS models (Figure 2). The prediction accuracy under a missing rate level of 0% was always the lowest for all traits as compared with other missing rate levels, and the prediction accuracy under the MAF level of 10% was the lowest for most traits as compared with other MAF levels. Steep slope was observed between prediction accuracies for missing rate levels of 0% and 20%. The major reason could be the significant difference of marker number between the two levels (Table 2). Stringent QC resulted in insufficient genome coverage and poor accuracy as well. However, prediction accuracy did not steadily increase with missing rate level (0% to 80%) and MAF level (0% to 10%). To find the best missing rate level, prediction accuracy under each missing rate was averaged across the seven GS models and three MAF levels for each trait. Considering the top three accuracies for each trait, missing rate levels 0%, 20% 40%, 60%, and 80% achieved the top accuracy for 0, 5, 5, 4, and 4 times, respectively, which indicated that, 20% to 40% was a suitable level for missing rate QC. Similarly, to find the best MAF level, prediction accuracy under each MAF level was averaged across the seven GS models and five missing rates for each trait (results not shown here). Considering the top two accuracies for each trait, MAF level 0%, 5%, and 10% achieved the top accuracy for 4, 5, and 3 times, respectively, which indicated that, 5% was a suitable level for MAF QC. In conclusion, missing rate levels of 20% to 40% and MAF level of 5% led to a suitable marker number and good or comparable prediction accuracies for all traits.

Prediction accuracy for the six traits and seven GS models with non-QC or QC to keep the missing rate levels <40% and MAF values >5% are shown in Table 3. The prediction accuracies for SN, SL, HD, and PH with QC were consistently higher than those with non-QC for all GS models, except LASSO for SN, RRBLUP for HD, and BLUP and RRBLUP for PH. The improvement was not significant for GY and TKW, except that LASSO for GY (Table 3). In this QC scenario, independent of the GS models, moderate prediction accuracies were observed for all traits (Table 3). The average prediction accuracy of GY, SN, TKW, SL, HD, and PH across all GS models was 0.522, 0.480, 0.601, 0.380, 0.350, and 0.572, which was partially related to trait heritability. For example, TKW and PH had a high heritability and high prediction accuracy, and SN and SL had a low heritability and low accuracy. However, GY had a low heritability but moderately high accuracy and HD had a high heritability but low accuracy. The possible reason could be the different genetic architectures of traits. For GY, variance of environment and genotype-by-environment interaction were similar, which were higher than genotypic variance, but for HD, environmental variance was much higher than the other two variances. The best models for GY, SN, TKW, SL, HD, and PH were LASSO, RForest, Moore–Penrose generalized inverse (GenInv), RidgeReg, GenInv (and also RidgeReg), and RidgeReg, respectively (Table 3). The average prediction accuracy of the seven GS models across the six traits was 0.489, 0.486, 0.512, 0.425, 0.494, 0.510, and 0.473, respectively. GenInv had the highest accuracy, followed by RidgeReg, RForest, BLUP, GBLUP, RRBLUP, and LASSO. Considering the top three accuracies for each trait, BLUP, GBLUP, GenInv, LASSO, RForest, RidgeReg, and RRBLUP achieved the top accuracy for 1, 2, 4, 1, 3, 5, and 2 times, respectively, which indicated that GenInv, RForest, and RidgeReg were relatively better among the seven models. In summary, independent of the GS models, moderate prediction accuracies were observed for all traits, and GenInv, RidgeReg, and RForest had a better performance than the other GS models for most traits (Table 3).

### 2.4. Effect of Imputation for Missing Genotypes on GS

The effect of genotype imputation on prediction accuracy was evaluated, using the QC to keep missing rate levels <40% and the MAF values >5% as an example. Prediction accuracies of yield and yield-related traits for the seven GS models using imputed markers are shown in Table 4. Compared with non-imputation (see Table 3 with QC), prediction accuracy using imputed markers sometimes increased slightly, but sometimes decreased slightly, irrespective of the traits and GS models (Table 4). Difference of prediction accuracy between imputation and non-imputation was minor. Averaged across the seven GS models, the prediction accuracy was 0.537, 0.496, 0.607, 0.423, 0.345, and 0.538 using imputed markers for GY, SN, TKW, SL, HD, and PH (last column in Table 4). Compared with results in Table 3, the accuracy improved by imputation for each trait was 0.015, 0.016, 0.006, 0.043, −0.005, and −0.034, respectively. Averaged across the six traits, the prediction accuracy using imputed markers was 0.472, 0.480, 0.521, 0.440, 0.509, 0.524, and 0.466 for the seven GS models, respectively (last row in Table 4). Compared with the results in Table 3, the accuracy improved by imputation for each model was −0.017, −0.006, 0.009, 0.015, 0.015, 0.014, and −0.007, respectively. The best models for the six traits were LASSO, RForest, GenInv (and also RidgeReg), RidgeReg, RidgeReg, and RForest, respectively (Table 4). Regarding the average performance across all traits, RidgeReg had the highest accuracy, followed by GenInv, RForest, GBLUP, BLUP, RRBLUP, and LASSO. Considering the top three accuracies for each trait, BLUP, GBLUP, GenInv, LASSO, RForest, RidgeReg, and RRBLUP achieved the top accuracy for 0, 2, 6, 1, 4, 6, and 0 times, respectively, which indicated that, GenInv, RidgeReg, and RForest were better among the seven models. In conclusion, imputation had marginal effect on GS, and it may not be a necessary step in the deployment of GS in wheat breeding. GenInv, RidgeReg, and RForest still had a better performance than the other GS models for most traits.

### 2.5. Effect of Significant Markers Detected by GWAS

Manhattan and quantile-quantile (Q-Q) plots from GWAS were given in Figure S3 for the imputed scenario, and in Figure S4 for the non-imputed scenario. The number of selected markers by GWAS is shown in Table 5. For both imputed and non-imputed scenarios, around 500 significant markers were detected by GWAS for each trait, which were, then, used for GS. For GWAS with imputed genotypic data, the number of selected markers was the highest for SN (537), and the lowest for PH (497). For GWAS with non-imputed genotypic data, the number of selected markers was also the highest for the SN (576), and the lowest for HD (506, Table 5). A comparison of Table 3 and Table 4 shows that using GWAS-derived markers in GS increased the prediction accuracy in most cases, irrespective of the traits and GS models. Conducting imputation before GWAS made a small increase on the prediction accuracy for most traits and GS models (Table 6). Averaged across the seven GS models, the prediction accuracy for the six traits was 0.847, 0.850, 0.873, 0.843, 0.793, and 0.798 under the imputed scenario, and was 0.785, 0.833, 0.843, 0.785, 0.800, and 0.803 under the non-imputed scenario (last column in Table 6). Average across the six traits, the prediction accuracy for the seven models was 0.913, 0.836, 0.895, 0.673, 0.711, 0.895, and 0.913 under the imputed scenario, and was 0.886, 0.792, 0.842, 0.737, 0.679, 0.836, and 0.886 under the non-imputed scenario (last row in Table 6). These values were much higher than those from GS without marker selection by GWAS (Table 3 and Table 4). The difference in accuracies between the imputed and non-imputed scenarios was minor. BLUP and RRBLUP had higher accuracy than the other models under both scenarios. Considering the top three accuracies for each trait, BLUP, GBLUP, GenInv, LASSO, RForest, RidgeReg, and RRBLUP achieved the top accuracy for 6, 1, 3, 0, 0, 4, and 6 times under the imputed scenario, and for 6, 0, 6, 0, 0, 2, and 6 times under the non-imputed scenario, respectively, which indicated that BLUP and RRBLUP were better among the seven models. The best models were different from those observed in Table 3 and Table 4. The reason could be that BLUP and RRLUP were more suitable for datasets with a small number of markers. In conclusion, using GWAS to select markers is a useful step for GS. Effect of genotype imputation before GWAS was very small. BLUP and RRBLUP had a better performance than the other GS models for most traits when GWAS-selected markers were used for GS.

## 3. Discussion

A better understanding of the factors that affect the prediction accuracy of GS is crucial to deploying GS within the conventional breeding scheme [12,19]. Missing rate and MAF are important factors that determine the quality of genotypic data. They have been extensively studied in animal breeding, but few such studies have been conducted in plant breeding [14,15,24]. According to the GS literatures, there is no consensus on marker QC thresholds for genomic prediction. Therefore, this study investigated the effect of missing rate and MAF QC on prediction accuracy for yield and yield-related traits in wheat. In addition, the effect of missing genotype imputation and GWAS-derived markers were also explored.

### 3.1. Marker Quality Control, Density, and LD

In most cases, QC on SNPs improved the prediction accuracy, irrespective of the traits and GS models. But the significance of improvement varied with trait features, GS models, as well as QC combinations. The QC levels for missing rate and MAF are important factors that affect prediction accuracy, in accordance with previous studies [15,24]. The increase in missing rate level resulted in an increased marker number, while the increase in MAF level resulted in a decreased marker number in the genome. The threshold of SNP QC can affect the quality of genotypic data, LD between markers and QTL, estimation of genetic relationship between individuals, and population structure [25]. Different genomic studies, including studies on QTL mapping, marker-assisted selection, and GWAS, have used different thresholds for marker QC [25]. In addition, different genotyping and sequencing platforms, such as SNP arrays and GBS (genotyping by sequencing), result in different qualities on genotypic data [26]. For instance, GBS is an effective genotyping technology that provides high marker density at a relatively low cost per sample, but it also generates a large proportion of missing data (up to 80%) when a low sequencing depth of genomic loci is employed [27]. However, markers with low MAF probably occur due to the design bias of the SNP array, because only a few cultivars and landraces are used to discover SNPs in the array [28].

Our results also revealed that QC for missing rate and MAF affected genome coverage (Table S2). The lowest number of markers was identified in the D genome, followed by the A and B genomes, which is in agreement with previous reports [23]. In addition, a stringent MAF threshold (e.g., >10% used in the present study) results in reduced allelic diversity in genomic datasets [29]. However, intrachromosomal LD decay declined rapidly with increasing distance (Figure S2). The LD decay rate is important because it determines the sufficient marker density for genome-wide coverage, i.e., at least one marker should be in LD with each segregating segment of the genome. In natural populations (non-inbred lines), faster LD decay requires higher marker density [30].

### 3.2. Effect of Missing Rate and MAF QC on Prediction Accuracy

Different missing rate thresholds have been adopted for QC on SNPs in previous studies [14,15,24,31]. However, it is difficult to determine which threshold is best for prediction accuracy. Therefore, QC with different missing rates and MAF thresholds was conducted to assess the predictability of the seven GS models in this study. Habier et al. [32] indicated that increasing marker density improves the genetic similarity of individuals in TP and VP, and thereby improves prediction accuracy. However, results from this study indicated that prediction accuracy was not improved consistently with an increase in missing rate level and MAF, irrespective of the traits (Figure 2). After QC for missing rate levels of 20% to 40%, all traits showed improved prediction accuracies, irrespective of the GS models (Figure 2). Including markers with a high missing rate level (e.g., 80%) added noise to the estimation of GEBVs. It is not necessary to use a small missing rate threshold (e.g., 0%), which undoubtedly reduces marker density and genome coverage, or even near to exclude some chromosomes (Table S2), reducing the prediction accuracy regardless of the GS models (Table 2 and Figure 2). The prediction accuracy was the highest when QC with a moderate missing data rate (20% to 40%) and MAF (5%) were used. Our conclusion was consistent with some previous studies. Roorkiwal et al. [33] evaluated prediction accuracy of six GS models for GS under nine combinations of missing rate and MAF QC for yield and yield-related traits in chickpea. Results indicated that QC on SNP before GS increased prediction accuracy, and missing rate levels ≤30% and MAF values ≥10% were the best QC combination. Jarquín et al. [15] compared different SNP QC (i.e., missing rate and MAF) scenarios in soybean and concluded that there was no unique strategy that outperformed the results of the others.

For most traits, prediction accuracies were negatively affected by a high MAF threshold (Figure 2). There were three possible reasons. First, the number of markers was smaller (insufficient genome coverage) and statistically less informative in the prediction analysis when a high MAF threshold was used (Table 2 and Table S2). Second, excluding markers by high MAF threshold could result in a bottleneck of allelic diversity [29] and biased accuracies for diverse germplasms. Third, excluded markers by QC can be linked with QTL, affecting some traits. In our diversity panel, it was possible that yield and yield-related traits were associated with relatively low-MAF SNPs which could have an advantage in the estimation of genetic relationship between TPs. The results from this study suggested that a moderate missing rate level (20% to 40%) and MAF (5%) threshold provided better prediction accuracy for yield and yield-related traits (Figure 2). There is no single combination of QC scenarios that outperforms the others for all traits and GS models. Further investigation is needed to determine how to find a balance between marker number and marker quality to achieve higher prediction accuracy. Further investigation is required to quantify the impact of other factors that were not included in this study such as TP size, population structure, and genotype-by-environment interactions, and imputation methods on prediction accuracy.

### 3.3. Effect of GS Models on Prediction Accuracy

The choice of GS models depends on the maximum prediction ability and computation efficiency of a model across a wide range of traits and datasets. In this study, the prediction accuracy varied substantially among traits and GS models (Table 3). There was no consistently best GS model for predicting various traits. This could be because the selected traits in the present study varied with respect to genetic architecture and heritability, whereas GS models differed from each other because of underlying assumptions for estimating marker effects [6,7]. In this study, GenInv, RidgeReg, and RForest had a higher prediction accuracy than other GS models (Table 3). The high accuracy of GenInv and RidgeReg could be explained by the overfitting of these two models, which was caused by the multi-collinearity between dense markers, overfitting results in biased estimation of marker effects, however, increasing the prediction accuracy in some cases. Another possible reason could be the trait features. Ornella et al. [34] reported the superiority of RidgeReg in some rust resistant traits because of the additive nature of rust resistance. The advantage of RForest is consistent with previous studies on wheat [18,19]. For example, Charmet et al. [10] compared the performances of five GS models on three elite breeding populations (each with approximately 350 lines) for three years and identified RForest to be the best model for predicting GY. Heslot et al. [7] evaluated seven GS models using eight datasets in wheat, barley, and maize, and demonstrated RF as a promising method to increase prediction accuracy. The superiority of RF for yield and yield-related was also reported in chickpea [33]. The superiority of RForest could be explained by its appealing properties for genomic predictions. RForest includes minor-effect QTLs and interacting and correlated markers with no distributional assumptions in the training model [8,35]. However, these three models may not show superiority in some other traits or populations. Further investigation is required to select the best GS model. In most cases, high-heritability traits result in high prediction accuracy, whereas low-heritability traits result in low prediction accuracy, regardless of the GS model [36]. Nevertheless, prediction accuracy relies not only on heritability but also on the genetic architecture of the target trait [37]. This phenomenon was also observed in this study.

### 3.4. Effect of Imputation and GWAS on Prediction Accuracy

The QC to keep missing rate levels <40% and MAF values >5% was used as an example to investigate the effect of imputation and GWAS on GS, and which QC scenario prediction accuracy was the highest. Missing values were imputed based on the empirical distribution of genotypes obtained from observed values, because this method requires less computational burden. Poland et al. [27] reported that imputation of missing values resulted in slightly higher prediction accuracy for yield (under drought condition), TKW, and HD, regardless of imputation methods (e.g., random forest, heterozygote, and expected maximization) in a panel of 254 advanced breeding lines from CIMMYT. Jarquín et al. [15] reported that imputation increased marker number and could improve prediction accuracy. However, in this study, it was concluded that imputation had little effect on the prediction accuracy of GS (Table 4), possibly because imputation and GS were conducted on the same dataset, and no additional information was provided to increase the prediction accuracy.

GWAS-derived markers improved prediction accuracy of GS, whether the imputation was conducted before GWAS or not. This was similar to the results by Lozada et al. [12], who reported that GS for GY using GWAS-derived markers had higher prediction accuracy than that using all markers in soft and red winter wheat. The possible reasons for the improvement in prediction accuracy included: (1) the number of GWAS-derived markers was smaller than the total marker number, which ultimately reduced multicollinearity and complexity of models for estimation of GEBVs; (2) the selected markers were all correlated with the traits. A low threshold (−log_10_ *P* = 1) was adopted in this study to identify more significant markers. The low threshold definitely increased the false positive of GWAS, but it is generally accepted that false positive does not have a significant effect on prediction accuracy. In this study, both TP and VP were used in GWAS, and GWAS and GS were conducted on the same population. In practice, the GS model constructed from TP would be used for other breeding populations only having genotypic data. More evidence is still needed before concluding that GWAS always improves the accuracy of GS.

## 4. Materials and Methods

### 4.1. Plant Materials, Field Trials, and Phenotypic Evaluation

The wheat population was comprised of 166 diverse accessions, namely, 143 accessions from the Yellow and Huai River Valley Facultative Wheat Zone of China and 23 varieties from five other countries (Argentina, Australia, Italy, Japan, and Turkey). The names and origins of these accessions are presented in Table S3. These accessions were grown in Anyang in Henan province for three cropping seasons (i.e., from 2013 to 2015), in Shangqiu for two seasons (i.e., 2013 and 2014), and in Shijiazhuang for one season (i.e., 2015). All field trials were conducted in a randomized complete block design. Each trial had three replications, and each plot had three rows that were 2 m in length and 0.2 m in width. The genotypic and phenotypic data used in this study can be downloaded from http://www.isbreeding.net/wheatGS/.

Six yield and yield-related traits, namely, GY, SN, TKW, SL, HD, and PH, were evaluated at each location. GY was measured as the weight of grain harvested kg·ha^−1^. SNs were counted for each plot and converted to spike number per square meter. TKW was measured by weighting 1000 random kernels from each plot after harvest. SLs were measured from the base of the rachis to the top spikelet, excluding awns. HD was recorded on 50% emergence of the spike; PH was measured as the distance between the soil surface and top of spike, excluding awns after physiological maturity. These traits were considered to represent a wide range of heritability and genetic architecture.

### 4.2. DNA Extraction, Genotyping, and Quality Control

Five fresh leaves of each accession were sampled, and DNA extraction was carried out by the modified CTAB method [38]. The genotypic data for the wheat accessions were obtained using a high-density Illumina 90K iSelect SNP array [39] featuring 81,587 SNPs. SNP genotyping was conducted by Genome Studio. A total of 21,856 SNPs remained for each accession using the genotypic data conversion function of QTL IciMapping V4.2 (freely available from https://www.isbreeding.net/) [40]. For QC on SNPs, the BIN function of QTL IciMapping v4.2 was used to remove the redundant markers, resulting in 14,043 non-redundant SNPs. Average missing rate of these markers was 28.20%. After binning, SNPs with more than 80% missing data were removed, and a total of 11,997 SNPs were employed to evaluate the prediction accuracy of the GS models.

### 4.3. Phenotypic Data Analysis and Analysis of Variance (ANOVA)

Descriptive statistics of phenotypic data were performed with Microsoft Excel 2016. Best linear unbiased estimates (BLUE) for each line across multiyear trials, ANOVA, and phenotypic correlation analysis were conducted using QTL IciMapping V4.2 [40]. BLUE values were used for correlation analysis. The ANOVA model across three locations is shown in Equation (1).

(1) $$y_{ijk}=\mu +R_{k/j}+G_{i}+E_{j}+GE_{ij}+\varepsilon_{ijk}\;\mathrm{and}\;\varepsilon_{ijk}\;\sim \;N\left(0,\sigma_{\varepsilon }^{2}\right)$$

where *y_ijk_* is the phenotypic value, *μ* is the overall mean, *G_i_* is the genotypic effect, *E_j_* is the environmental effect, *GE_ij_* is the genotype-by-environment interaction effect, *R_k/j_* is the *k*th replication effect in the *j*th environment, and *ε_ijk_* is the residual effect. From the theoretical expectation of mean square (MS), genetic variance ($\sigma_{G}^{2}$), interaction variance ($\sigma_{GE}^{2}$), and error variance ($\sigma_{\varepsilon }^{2}$) were calculated using Equation (2), where environment number *e* = 3, and replication number *r* = 3 in the present study.

(2) $$\sigma_{G}^{2}=\frac{1}{e\times r}\left(MS_{G}-MS_{\varepsilon }\right),\;\sigma_{GE}^{2}=\frac{1}{r}\left(MS_{GE}-MS_{\varepsilon }\right),\;\mathrm{and}\;\sigma_{\varepsilon }^{2}=MS_{\varepsilon }$$

Heritability at the plot level and mean level was calculated using Equation (3).

(3) $$H_{\mathrm{perplot}}^{2}\;=\;\frac{\sigma_{G}^{2}}{\sigma_{G}^{2}+\sigma_{GE}^{2}+\sigma_{\varepsilon }^{2}}\;\mathrm{and}\;H_{\mathrm{per}\;\mathrm{mean}}^{2}\;=\;\frac{\sigma_{G}^{2}}{\sigma_{G}^{2}+\frac{1}{e}\sigma_{GE}^{2}+\frac{1}{er}\sigma_{\varepsilon }^{2}}$$

### 4.4. Genotypic Data Analysis

LD between markers measured as *r*^2^ was calculated by the TASSEL software (freely available from https://tassel.bitbucket.io/) using the full matrix and sliding window options [41] and plotted against physical distance. Markers with a missing rate lower than 40% were used for LD analysis. The PIC and GD (also known as expected heterozygosity) at each locus were estimated using PowerMarker v.3.5 (freely available from https://brcwebportal.cos.ncsu.edu/powermarker/) [42]. Plot visualizations of these parameters were generated by the ggplot2 package in R (freely available from https://cran.r-project.org/web/packages/ggplot2/index.html) [43].

### 4.5. GS Models and Factors Affecting Prediction Accuracy

Seven GS models were implemented in the Intel FORTRAN program for estimating prediction accuracies (code written by L.Z. and J.W.), i.e., BLUP [44], GBLUP [45], RRBLUP [46], RidgeReg, GenInv, LASSO [47], and one machine learning method, i.e., RForest [8]. Five-fold cross-validation was employed and replicated 50 times to avoid biases in the estimation of prediction accuracy. The averaged prediction accuracy across the 50 replicates was calculated. To assess the impact of the missing rate and MAF on prediction accuracy, five missing rate thresholds (i.e., 0%, <20%, <40%, <60%, and <80%) and three MAF thresholds (i.e., >0%, >5%, and >10%) were considered. This produced 15 marker datasets (e.g., 5 missing marker levels × 3 MAF levels). Prediction accuracy was evaluated in terms of Pearson’s correlation between the observed adjusted phenotypic values (i.e., BLUE) and predicted values (i.e., GEBVs). In order to investigate the effect of QC on GS, QC with missing rate levels <40% and MAF values >5% was used as an example, and compared with non-QC, in which all polymorphic markers were used. *T*-test was conducted to compare the significance of difference between QC and non-QC cases. The detailed descriptions of these seven models are described in the next subsection. Comparison of prediction accuracy among traits and GS models was also conducted under this QC scenario, i.e., missing rate levels <40% and MAF >5%.

#### 4.5.1. BLUP Model

The BLUP mixed model is described as follows:

(4) y=Xβ+Zu+ε

where *y* is a (*n* × 1) vector of phenotypic values; *X* is a (*n* × *p*) incidence matrix for fixed effects; *β* is a (*p* × 1) vector of fixed effects; *Z* is a (*n* × 1) incidence matrix for random effects; u is a vector of random effects; *ε* is a (*n* × 1) vector of independently random residual following distribution $N\left(0,I\sigma_{u}^{2}\right)$, with *I* being the identity matrix in the present case [44]. In addition, *p* is the number of fixed effects, *n* is the number of genotypes, and *m* is the number of markers.

#### 4.5.2. GBLUP Model

The standard GBLUP model is described as follows:

(5) $$y=1_{n}\mu +Zu+\varepsilon$$

where *y* is the vector of phenotypic values, 1*_n_* is the vector of *n* ones, *μ* is the overall mean, *Z* is the design matrix for random effects, *u* is the random effect with $u\sim N\left(0,G\sigma_{u}^{2}\right)$, *G* is a genomic relationship matrix between individuals estimated from genotypes, and *ε* is the vector of random residuals following distribution $N\left(0,I\sigma_{\varepsilon }^{2}\right)$ [45,46].

#### 4.5.3. RRBLUP Model

The rrBLUP model is described as follows:

(6) $$y=1_{n}\mu +Zu+\varepsilon$$

where *y* is the vector of phenotypic values; 1*_n_* is the vector of *n* ones; *μ* is the overall mean; Z is the design matrix for random effects; *u* is the random e1ffect with $u\sim N\left(0,K\sigma_{u}^{2}\right)$; *K* is the additive relationship matrix, which is the density matrix in the present study; and *ε* is the vector of random residuals following distribution $N\left(0,I\sigma_{\varepsilon }^{2}\right)$ [46].

#### 4.5.4. RidgeReg Model

The model is fitted by:

(7) y=Xβ+Zu+ε

where *y* is the vector of phenotypic values, X is the design matrix for fixed effects, *β* is the vector of marker fixed effects, Z is the design matrix for random effects, *u* is the vector of random effects, *ε* is the vector of random residual following distribution $N\left(0,I\sigma_{u}^{2}\right)$, and *I* is the identity matrix. The estimator of *β* is ${\left(X^{\prime }X+\lambda I\right)}^{-1}X^{\prime }y$, and the estimator of *λ* can be expressed as $\underset{\beta }{\arg\;\min}(\|y-X\beta \|{}_{2}^{2}+\lambda {\left\|\beta \right\|}_{2}^{2})$, with the notation ${\left\|.\right\|}_{2}$ for L_2_ norm. The “$\underset{\beta }{\arg\;\min}$” notation expresses the determination of coefficient *β* minimizing the expression inside the brackets.

#### 4.5.5. GenInv Model

The model is the same as the model described in Section 4.5.4, but the estimation of *β* is ${\left(X^{\prime }X\right)}^{+}X^{\prime }y$. Here, “+” is the Moore–Penrose generalized inverse of matrix.

#### 4.5.6. LASSO Model

The LASSO was developed by Tibshirani [47]. *λ* refers to the shrinkage and regularization parameter calculated by $\underset{\beta }{\arg\;\min}(\frac{\|y-X\beta \|{}_{2}^{2}}{2\sigma^{2}}+\lambda {\left\|\beta \right\|}_{1})$ and $\left\|\beta \right\|=\sum_{i}\left\|\beta_{i}\right\|$ is the L_1_ norm.

#### 4.5.7. RForest Model

The RForest model is integrated with classification or regression trees that rely on bootstrap samples and splits original data into multiple subsets of non-overlapping sets [16]. The predictions for observations are calculated as the averages of predicted values over the trees.

### 4.6. Imputation for Missing Genotypes

To evaluate the effect of genotype imputation on prediction accuracy of GS, missing values in genotypic data were imputed using samples from the empirical distribution of marker genotypes [48]. Imputed markers were employed for genomic prediction analysis. The QC to keep missing rate levels <40% and MAF values >5% was used as an example, i.e., 5513 markers were retained after QC and employed for imputation and GS.

### 4.7. GWAS-Derived Genomic Selection

To evaluate the effect of GWAS-based marker selection on prediction accuracy of GS, the “GWAS” function of R package rrBLUP V4.6 (freely available from https://cran.r-project.org/web/packages/rrBLUP/index.html/) was used to perform GWAS before GS [46]. To avoid spurious marker-trait associations due to population structure, a realized additive relationship matrix and the first five principal components were included in the model (i.e., PCA + K model). The additive relationship matrix was computed using “A.mat” function of rrBLUP. Manhattan plots were also generated by rrBLUP [46]. The threshold of −log_10_ *P* was set at 1, in order not to miss any small-effect quantitative trait nucleotides. The QC to keep missing rate levels <40% and MAF values >5% was used as an example. Markers with unknown physical positions were excluded before GWAS. As a result, a total of 5201 markers were employed for GWAS. Two GWAS-derived GS scenarios were designed. In the first scenario, imputation for missing genotypes was conducted before GWAS; in the second scenario, non-imputed genotypic data was used to perform GWAS.

## 5. Conclusions

In this study, a diverse Chinese winter wheat panel was used to compare prediction accuracy of seven GS models (BLUP, GBLUP, GenInv, LASSO, RForest, RidgeReg, and RRBLUP) under different marker QC scenarios (five missing rate levels and three MAF values) for yield and yield-related traits. No single QC combination or GS model can yield better performance in prediction accuracy for all traits. In general, a combination of moderate missing rate levels (20% to 40%) and MAF (5%) yielded better prediction accuracy, regardless of the traits and GS models. Prediction accuracy of the six traits was affected by the heritability, genetic architecture, and GS models. GenInv, RidgeReg, and RForest models yielded higher prediction accuracy than other models across traits. The effect of genotype imputation and GWAS-based marker selection was also evaluated in this study. The results showed that imputation had marginal effect on GS but using GWAS-derived markers improved the prediction accuracy of GS.

## Figures and Tables

**Table 1:** Variance components and heritability of yield and yield-related traits in 166 wheat accessions.

Trait ^1^	Variance Components (%)				Heritability ^3^	
	Genotype	Environment	G by E Interaction ^2^	Random Error	Plot Level	Genotypic Mean Level
GY	12.12	43.00	39.39	5.50	0.42	0.85
SN	34.32	24.11	36.19	5.39	0.69	0.92
TKW	41.38	27.68	23.76	7.18	0.77	0.97
SL	42.94	8.24	34.71	14.12	0.67	0.96
HD	12.64	79.29	7.26	0.81	0.81	0.97
PH	60.19	11.97	23.09	4.75	0.85	0.98

^1^ GY, grain yield; SN, spike number per square meter; TKW, thousand-kernel weight; SL, spike length; HD, heading days; PH, plant height. ^2^ G by E; genotype-by-environment. ^3^ Heritability was estimated from analysis of variance across environments.

**Table 2:** Number of markers used for genomic predictions under five missing rate levels (i.e., 0%, <20%, <40%, <60%, and <80%) and three minor allele frequency (MAF) levels (i.e., >0%, >5%, and >10%).

Missing Rate (%)	MAF (%)		
	0 ^1^	5	10
0 ^2^	1442	259	181
20	8674	5343	4368
40	9851	5513	4494
60	10818	5635	4596
80	11997	5725	4675

^1^ MAF greater than zero. In other words, this QC scenario actually only removed non-polymorphism markers in the population, and therefore the remaining markers were polymorphic after this control. ^2^ Markers contained no missing values.

**Table 3:** Prediction accuracy with marker quality control (QC) or non-QC for six traits and seven genomic selection (GS) models. The QC to keep markers with missing rate levels <40% and minor allele frequency (MAF) >5% was used as an example.

Trait ^1^	Scenario	Genomic Selection Model ^2^							
		BLUP	GBLUP	GenInv	LASSO	RForest	RidgeReg	RRBLUP	Mean
GY	QC	0.531 (0.027) ^3^	0.458 (0.033)	0.503 (0.029)	**0.589**^5^ (0.022)	**0.547** (0.028)	0.495 (0.030)	**0.534** (0.027)	0.522 (0.016)
	Non-QC ^4^	0.545 (0.030)	0.461 (0.035)	0.506 (0.033)	0.454 (0.029)	0.535 (0.028)	0.506 (0.033)	0.545 (0.030)	0.507 (0.014)
	*p*-value	0.3687	0.3892	0.3926	0.0001	0.3688	0.4662	0.3994	0.248
SN	QC	0.491 (0.026)	0.484 (0.025)	**0.496** (0.025)	0.383 (0.030)	**0.521** (0.027)	**0.494** (0.025)	0.488 (0.026)	0.480 (0.017)
	Non-QC	0.462 (0.030)	0.383 (0.034)	0.335 (0.035)	0.444 (0.033)	0.434 (0.031)	0.335 (0.035)	0.463 (0.031)	0.408 (0.021)
	*p*-value	0.2447	0.0096	0.0002	0.0816	0.018	0.0002	0.2671	0.011
TKW	QC	0.600 (0.028)	**0.605** (0.038)	**0.652** (0.027)	0.499 (0.036)	0.603 (0.028)	**0.650** (0.028)	0.601 (0.028)	0.601 (0.019)
	Non-QC	0.672 (0.025)	0.598 (0.032)	0.619 (0.026)	0.594 (0.027)	0.638 (0.027)	0.619 (0.026)	0.672 (0.025)	0.630 (0.012)
	*p*-value	0.0282	0.4463	0.19	0.0189	0.1809	0.2122	0.0298	0.116
SL	QC	0.373 (0.046)	**0.402** (0.041)	**0.416** (0.041)	0.315 (0.041)	0.373 (0.040)	**0.417** (0.040)	0.362 (0.050)	0.380 (0.014)
	Non-QC	0.307 (0.047)	0.367 (0.042)	0.358 (0.042)	0.146 (0.047)	0.284 (0.042)	0.358 (0.042)	0.296 (0.048)	0.302 (0.029)
	*p*-value	0.1595	0.2805	0.1629	0.0041	0.0645	0.1575	0.1721	0.020
HD	QC	0.355 (0.038)	0.394 (0.034)	**0.413** (0.033)	0.264 (0.033)	**0.342** (0.033)	**0.413** (0.033)	0.266 (0.040)	0.350 (0.024)
	Non-QC	0.326 (0.036)	0.276 (0.036)	0.262 (0.040)	0.161 (0.047)	0.340 (0.027)	0.262 (0.040)	0.272 (0.033)	0.271 (0.022)
	*p*-value	0.2912	0.0265	0.0065	0.0412	0.471	0.0063	0.4991	0.017
PH	QC	**0.585** (0.019)	0.570 (0.020)	0.593 (0.019)	0.502 (0.032)	0.576 (0.027)	**0.591** (0.020)	**0.586** (0.019)	0.572 (0.012)
	Non-QC	0.616 (0.022)	0.543 (0.030)	0.574 (0.023)	0.369 (0.039)	0.558 (0.027)	0.574 (0.040)	0.615 (0.033)	0.550 (0.032)
	*p*-value	0.1438	0.2283	0.2445	0.0057	0.3201	0.2807	0.1372	0.269
Mean	QC	0.489 (0.043)	0.486 (0.035)	0.512 (0.039)	0.425 (0.051)	0.494 (0.045)	0.510 (0.039)	0.473 (0.054)	
	Non-QC	0.488 (0.061)	0.438 (0.049)	0.442 (0.059)	0.461 (0.072)	0.465 (0.056)	0.442 (0.059)	0.477 (0.067)	

^1^ GY, grain yield; SN, spike number per square meter; TKW, thousand-kernel weight; SL, spike length; HD, heading days; PH, plant height. ^2^ BLUP, best linear unbiased prediction; GBLUP, genomic-BLUP; GenInv, Moore–Penrose generalized inverse; LASSO, least absolute shrinkage and selection operator; RForest, random forest; RidgeReg, ridge regression; and RRBLUP, ridge regression-BLUP. ^3^ Values in parenthesis indicate standard errors of the estimated parameter. ^4^ Non-QC indicates that all polymorphic markers were used. ^5^ The models with the top three prediction accuracies under QC are bolded for each trait.

**Table 4:** Prediction accuracies of yield and yield-related traits for the seven GS models using imputed markers. The QC to keep markers with missing rate levels <40% and minor allele frequency (MAF) >5% was used as an example.

Trait ^1^	Genomic selection Model ^2^							
	BLUP	GBLUP	GenInv	LASSO	RForest	RidgeReg	RRBLUP	Mean
GY	0.517 (0.031) ^3^	0.491 (0.031)	**0.531**^4^ (0.024)	**0.593** (0.022)	**0.577** (0.024)	**0.531** (0.024)	0.520 (0.031)	0.537 (0.13)
SN	0.488 (0.025)	0.477 (0.036)	**0.520** (0.024)	0.418 (0.029)	**0.569** (0.026)	**0.518** (0.029)	0.481 (0.026)	0.496 (0.018)
TKW	0.600 (0.036)	0.560 (0.043)	**0.636** (0.024)	0.586 (0.031)	**0.629** (0.03)	**0.636** (0.031)	0.602 (0.035)	0.607 (0.011)
SL	0.370 (0.04)	**0.455** (0.041)	**0.489** (0.024)	0.370 (0.034)	0.394 (0.036)	**0.494** (0.036)	0.392 (0.044)	0.423 (0.021)
HD	0.305 (0.031)	**0.380** (0.030)	**0.381** (0.024)	0.377 (0.034)	0.312 (0.057)	**0.401** (0.029)	0.256 (0.035)	0.345 (0.02)
PH	0.549 (0.019)	0.517 (0.0241)	**0.566** (0.024)	0.450 (0.035)	**0.570** (0.025)	**0.566** (0.02)	0.547 (0.02)	0.538 (0.016)
Mean	0.472 (0.046)	0.480 (0.025)	0.521 (0.036)	0.440 (0.036)	0.509 (0.051)	0.524 (0.051)	0.466 (0.051)	

^1^ GY, grain yield; SN, spike number per square meter; TKW, thousand-kernel weight; SL, spike length; HD, heading days; PH, plant height; ^2^ BLUP, best linear unbiased prediction; GBLUP, genomic-BLUP; GenInv, Moore–Penrose generalized inverse; LASSO, least absolute shrinkage and selection operator; RForest, random forest; RidgeReg, ridge regression; and RRBLUP, ridge regression-BLUP. ^3^ Values in parenthesis indicate standard errors of the estimated parameter. ^4^ The models with the top three prediction accuracies with and without markers imputation are bolded for each trait.

**Table 5:** The number of significant markers detected by genome-wide association studies (GWAS) under the imputed and non-imputed scenarios. Threshold of −log_10_ *P* was set at 1.

Trait ^1^	GWAS QTLs	
	Imputed ^2^	Non-imputed
GY	525	514
SN	537	576
TKW	519	553
SL	520	509
HD	508	506
PH	497	522
Total	3106	3080

^1^ GY, grain yield; SN, spike number per square meter; TKW, thousand-grain weight; SL, spike length; HD, heading days; PH, plant height. ^2^ Markers were imputed before GWAS.

**Table 6:** Prediction accuracy for yield and yield-related traits using significant markers detected by genome-wide association studies (GWAS). Both imputed and non-imputed scenarios were considered. The QC to keep markers with missing rate levels <40% and minor allele frequency (MAF) >5% was used as an example.

Trait ^1^	Imputation ^2^	Genomic Selection Model ^3^							
		BLUP	GBLUP	GenInv	LASSO	RForest	RidgeReg	RRBLUP	Mean
GY	Yes	**0.901**^4^ (0.008) ^5^	**0.901** (0.008)	0.887 (0.008)	0.721 (0.016)	0.730 (0.020)	0.887 (0.008)	**0.901** (0.008)	0.847 (0.031)
	No	**0.866** (0.01)	0.712 (0.038)	**0.788** (0.014)	0.746 (0.017)	0.727 (0.017)	**0.788** (0.014)	**0.866** (0.010)	0.785 (0.024)
SN	Yes	**0.923** (0.007)	0.859 (0.037)	**0.915** (0.007)	0.673 (0.025)	0.741 (0.018)	**0.915** (0.007)	**0.923** (0.005)	0.850 (0.039)
	No	**0.889** (0.011)	0.857 (0.018)	**0.872** (0.010)	0.767 (0.016)	0.685 (0.023)	**0.872** (0.010)	**0.889** (0.011)	0.833 (0.029)
TKW	Yes	**0.942** (0.006)	0.848 (0.034)	**0.938** (0.005)	0.737 (0.024)	0.763 (0.021)	0.938 (0.005)	**0.942** (0.007)	0.873 (0.034)
	No	**0.926** (0.005)	0.79 (0.036)	**0.881** (0.010)	0.763 (0.018)	0.733 (0.021)	0.880 (0.010)	**0.926** (0.005)	0.843 (0.03)
SL	Yes	**0.932** (0.005)	0.861 (0.034)	0.938 (0.005)	0.628 (0.026)	0.674 (0.029)	**0.938** (0.005)	**0.932** (0.005)	0.843 (0.051)
	No	**0.881** (0.012)	0.773 (0.044)	**0.841** (0.016)	0.669 (0.029)	0.613 (0.034)	0.840 (0.016)	**0.881** (0.012)	0.785 (0.04)
HD	Yes	**0.878** (0.011)	0.792 (0.030)	0.846 (0.012)	0.660 (0.026)	0.648 (0.024)	**0.846** (0.002)	**0.878** (0.011)	0.793 (0.037)
	No	**0.873** (0.010)	0.818 (0.021)	**0.827** (0.013)	0.728 (0.018)	0.653 (0.020)	0.826 (0.013)	**0.873** (0.010)	0.800 (0.031)
PH	Yes	**0.901** (0.008)	0.756 (0.038)	**0.846** (0.013)	0.621 (0.029)	0.712 (0.024)	**0.846** (0.013)	**0.901** (0.008)	0.798 (0.040)
	No	**0.880** (0.011)	0.800 (0.023)	**0.840** (0.017)	0.746 (0.023)	0.664 (0.020)	0.810 (0.017)	**0.880** (0.011)	0.803 (0.029)
Mean	Yes	0.913 (0.010)	0.836 (0.021)	0.895 (0.017)	0.673 (0.019)	0.711 (0.018)	0.895 (0.017)	0.913 (0.100)	
	No	**0.886** (0.009)	0.792 (0.020)	**0.842** (0.014)	0.737 (0.015)	0.679 (0.019)	0.836 (0.019)	**0.886** (0.009)	

^1^ GY, grain yield; SN, spike number per square meter; TKW, thousand-kernel weight; SL, spike length; HD, heading days; PH, plant height. ^2^ “Yes” indicates that genotypic data was imputed for missing values and then used for GWAS and GS analysis. ^3^ BLUP, best linear unbiased prediction; GBLUP, genomic-BLUP; GenInv, Moore–Penrose generalized inverse; LASSO, least absolute shrinkage and selection operator; RForest, random forest; RidgeReg, ridge regression; and RRBLUP, ridge regression-BLUP. ^4^ The models with the top three prediction accuracies with and without markers imputation are bolded for each trait. ^5^ Values in parenthesis indicate standard errors of the estimated parameter.

## Supplementary Materials

Supplementary Materials can be found at https://www.mdpi.com/1422-0067/21/4/1342/s1.

## Abbreviations

ANOVA	Analysis of variance
BLUE	Best linear unbiased estimates
BLUP	Best linear unbiased predictors
GEBV	Genomic estimated breeding values
GenInv	Moore-Penrose generalized inverse
GBS	Genotyping by sequencing
GS	Genomic selection
GWAS	Genome-wide association studies
GY	Grain yield
HD	Heading days
LASSO	Least absolute shrinkage and selection operator
LD	Linkage disequilibrium
MAF	Minor allele frequency
PH	Plant height
QC	Quality control
RForest	Random forest
RidgeReg	Ridge regression
RRBLUP	Ridge regression best linear unbiased predictors
SL	Spike length
SN	Spike number
SNP	Single nucleotide polymorphism
TKW	Thousand-kernel weight
TP	Training population
VP	Validating population
