# Supplementary material for: Genomic Prediction for Grain Yield and Yield-Related Traits in Chinese Winter Wheat
Source: Int J Mol Sci. 2020 Feb 17;21(4):1342. doi: 10.3390/ijms21041342 (PMC7073225; doi:10.3390/ijms21041342)
Supplement: Supplementary file 1 [file ijms-21-01342-s001.pdf]

Genomic Prediction for Grain Yield and Yield-Related Traits in Chinese Winter Wheat

Mohsin Ali [1], Yong Zhang [1], Awais Rasheed [2,3], Jiankang Wang [1] and Luyan Zhang [1,*]

[1] National Key Facility for Crop Gene Resources and Genetic Improvement, and Institute of Crop Sciences, Chinese Academy of Agricultural Sciences (CAAS), Beijing 100081, China.; mali1990@yahoo.com (M.A.); zhangyong05@caas.cn (Y.Z.); wangjiankang@caas.cn (J.W.)

[2] International Maize and Wheat Improvement Center (CIMMYT) China Office, c/o CAAS, 12 Zhongguancun South Street, Beijing 100081, China; arasheed@qau.edu.pk

[3] Department of Plant Sciences, Quaid-i-Azam University, Islamabad 45320, Pakistan

* Correspondence: zhangluyan@caas.cn; Tel.: +86-10-82108572

Supplementary figures and tables

Table S1. Summary statistics of yield and yield-related traits for the 166 wheat accessions in six environments.

Trait [1]	Cropping season	Min	Max	Mean	Variance	Heritability	
						Plot level	Mean level
GY	Anyang (2012-2013)	6267.85	12488.89	9318.19	1574238.93	0.75	0.90
	Shangqiu (2012-2013)	3068.44	9074.07	6320.25	1169743.31	0.79	0.92
	Anyang (2013-2014)	5859.77	12547.11	9178	1507951.65	0.76	0.91
	Shangqiu (2013-2014)	5065.33	11373.78	8562.35	1330662.32	0.78	0.91
	Anyang (2014-2015)	4677.78	9355	7328.31	1196005.38	0.88	0.96
	Shijiazhuang (2014-2015)	3638.88	7683.33	6155.99	729754.45	0.87	0.95
	BLUE [2]	5090.46	8535.81	7268.81	548010.17		
SN	Anyang (2012-2013)	356	943	693	12150	0.89	0.96
	Shangqiu (2012-2013)	276	804	534	9395	0.88	0.96
	Anyang (2013-2014)	291	846	578	7633	0.88	0.96
	Shangqiu (2013-2014)	220.44	892	632	8488	0.89	0.96
	Anyang (2014-2015)	NA [3]	NA	NA	NA	NA	NA
	Shijiazhuang (2014-2015)	NA	NA	NA	NA	NA	NA
	BLUE	297	834	605	6680		
TKW	Anyang (2012-2013)	25.26	56.09	41.14	29.06	0.90	0.97
	Shangqiu (2012-2013)	25.67	56.26	42.16	36.25	0.83	0.93
	Anyang (2013-2014)	28.33	63.42	49.82	32.02	0.93	0.98
	Shangqiu (2013-2014)	22.50	62.166	45.84	42.60	0.80	0.92
	Anyang (2014-2015)	24.77	50.66	39.38	25.85	0.85	0.94
	Shijiazhuang (2014-2015)	22.95	49.98	39.64	27.02	0.92	0.97
	BLUE	25.66	54.51	43.17	24.49		
SL	Anyang (2012-2013)	6.83	12.66	8.94	1.00	0.81	0.93
	Shangqiu (2012-2013)	7.93	13.73	9.64	1.11	0.70	0.88
	Anyang (2013-2014)	6.70	11.40	8.83	1.02	0.77	0.91
	Shangqiu (2013-2014)	7.23	12.40	9.54	1.33	0.77	0.91
	Anyang (2014-2015)	7.64	12.82	9.58	1.08	0.73	0.89
	Shijiazhuang (2014-2015)	7.24	12.78	8.93	0.94	0.88	0.96
	BLUE	7.36	12.33	9.15	0.74		
HD	Anyang (2012-2013)	190	208	198	5.20	0.93	0.97
	Shangqiu (2012-2013)	184	203	189	11.42	0.93	0.98
	Anyang (2013-2014)	177	201	185	11.18	1.00	1.00
	Shangqiu (2013-2014)	178	201	184	9.79	0.93	0.98
	Anyang (2014-2015)	188	206	195	5.78	0.94	0.98

	Shijiazhuang (2014-2015)	195	215	199	5.36	0.93	0.98
	BLUE	179	202	187	9.68		
PH	Anyang (2012-2013)	63.33	145	84.10	165.73	0.96	0.99
	Shangqiu (2012-2013)	55	133.33	80.11	135.21	0.93	0.98
	Anyang (2013-2014)	58.23	135.66	77.60	171.62	0.91	0.97
	Shangqiu (2013-2014)	59.76	130.13	82.89	121.44	0.94	0.98
	Anyang (2014-2015)	69.86	136.26	91.52	108.00	0.92	0.97
	Shijiazhuang (2014-2015)	63.43	120.09	81.75	93.41	0.93	0.98
	BLUE	62.59	132.39	83.36	112.06		

[1] GY, grain yield ($Kg \cdot ha^{-1}$); SN, spike number per square meter; TKW, thousand-grain weight (g); SL, spike length (cm); HD, heading days (d); PH, plant height (cm). [2] BLUE, best linear unbiased estimation. [3] No observation in the environment.

Table S2. Distribution of markers used for genomic selection under five missing marker rate (underlined; %) levels (i.e., 0, < 20, < 40, < 60, and < 80%) and three minor allele frequency (MAF; %) levels (i.e., > 0, > 5, and > 10%).

**Chr.**	**0% MAF**					**5% MAF**					**10% MAF**				
	**<u>0</u>**	**<u>20</u>**	**<u>40</u>**	**<u>60</u>**	**<u>80</u>**	**<u>0</u>**	**<u>20</u>**	**<u>40</u>**	**<u>60</u>**	**<u>80</u>**	**<u>0</u>**	**<u>20</u>**	**<u>40</u>**	**<u>60</u>**	**<u>80</u>**
1A	37	254	289	323	368	18	331	339	350	355	11	261	266	277	282
1B	45	333	375	409	448	14	300	308	312	320	14	249	254	258	264
1D	41	236	276	292	317	1	170	175	177	179	1	138	141	143	145
2A	64	319	360	392	433	27	368	383	391	401	18	293	305	309	317
2B	62	448	506	566	635	22	374	385	392	397	16	292	299	303	306
2D	46	257	287	306	349	8	177	183	188	191	4	142	148	152	153
3A	53	290	327	366	407	15	325	336	342	346	11	267	275	281	285
3B	54	328	373	399	439	16	315	325	327	330	13	260	270	271	274
3D	32	169	197	225	252	11	132	133	138	143	9	112	113	118	122
4A	47	229	249	280	311	11	284	288	292	294	5	235	237	241	243
4B	32	211	245	274	310	9	184	192	196	199	6	160	167	171	174
4D	24	124	140	152	172	8	84	87	89	90	6	65	66	68	68
5A	43	267	310	348	375	11	279	288	297	299	6	230	234	240	244
5B	57	366	415	454	496	7	308	321	328	335	4	249	262	268	275
5D	48	229	252	276	308	10	140	143	147	148	5	114	116	120	120
6A	46	242	287	310	346	13	259	268	273	277	10	222	228	233	236
6B	47	287	326	359	393	13	244	252	256	261	10	198	204	208	212
6D	31	155	176	195	210	5	91	94	97	99	3	76	78	81	83
7A	50	312	365	407	444	15	339	354	367	376	12	284	296	305	314
7B	36	291	336	368	419	6	233	243	251	256	4	190	196	203	208
7D	34	219	250	265	293	7	129	133	135	138	5	110	113	116	119
Unmapped SNP	513	3108	3510	3852	4272	12	277	283	290	291	8	221	226	230	231
A genome	340	1913	2187	2426	2684	110	2185	2256	2312	2348	73	1792	1841	1886	1921
B genome	333	2264	2576	2829	3140	87	1958	2026	2062	2098	67	1598	1652	1682	1713
D genome	256	1389	1578	1711	1901	50	923	948	971	988	33	757	775	798	810
Total SNP	1442	8674	9851	10818	11997	259	5343	5513	5635	5725	181	4368	4494	4596	4675

Table S3. The 166 wheat accessions used in genomic prediction for yield and related traits.

No.	Name	Origin
1	11CA40	Henan
2	85Zhong 33	Henan
3	Aca 601	Argentina
4	Aca 801	Argentina
5	Barra	Italy
6	Dorico	Italy
7	Genio	Italy
8	HK1/6/NVSR3/5/BEZ/TVR/5/CFN/BEZ//SU92/CI13645/3NAI60	Turkey
9	Kanto 107	Japan
10	Kitanokaori	Japan
11	Klein Flecha	Argentina
12	Klein Jabal1	Argentina
13	Lampo	Italy
14	Libero	Italy
15	Mantol	Italy
16	Nidera Baguette 10	Argentina
17	Nidera Baguette 20	Argentina
18	Norin 61	Japan
19	Norin 67	Japan
20	PH 82-2	Shandong
21	Prointa Colibr 1	Argentina
22	Sagittario	Italy
23	Sunstate	Australia
24	Abbondanza	Italy
25	Funo	Italy
26	Aifeng 3	Shaanxi
27	Aikang 58	Henan
28	An 1331	Anhui
29	Bainong 3217	Henan
30	Bainong 64	Henan
31	Bima 1	Shaanxi
32	Bima 4	Shaanxi
33	Fengchan 3	Shaanxi
34	Fu 936	Anhui
35	Gaoyou 503	Hebei
36	Gaocheng 8901	Hebei
37	Hengguan 35	Hebei

38	Han 6172	Hebei
39	Heng 7228	Hebei
40	Hengguan 33	Hebei
41	Huapei 5	Henan
42	Huaimai 18	Jiangsu
43	Huaimai 20	Jiangsu
44	Huaimai 21	Jiangsu
45	Jimai 19	Shandong
46	Jimai 20	Shandong
47	Jimai 21	Shandong
48	Jimai 22	Shandong
49	Jinan 13	Shandong
50	Jinan 17	Shandong
51	Jining 16	Shandong
52	Shiluan 02-1	Hebei
53	Jinhe 9123	Hebei
54	Jinmai 61	Shanxi
55	Lankao 24	Henan
56	Lankao 2	Henan
57	Lankao 906	Henan
58	Liangxing 66	Shandong
59	Liangxing 99	Shandong
60	Linhan 2	Shanxi
61	Linkang 12	Shanxi
62	Linmai 2	Shandong
63	Linmai 4	Shandong
64	Lumai 11	Shandong
65	Lumai 14	Shandong
66	Luami 15	Shandong
67	Lumai 21	Shandong
68	Lumai 23	Shandong
69	Lumai 5	Shandong
70	Lumai 6	Shandong
71	Lumai 7	Shandong
72	Lumai 8	Shandong
73	Lumai 9	Shandong
74	Luyuan 502	Shandong
75	Luohan 2	Henan
76	Luomai 21	Henan
77	Neixiang 188	Henan
78	Neixiang 5	Henan

79	Shannong 20	Shandong
80	Shaan 150	Shaanxi
81	Shaan 229	Shaanxi
82	Shaan 253	Shaanxi
83	Shaan 354	Shaanxi
84	Shaan 512	Shaanxi
85	Shaan 715	Shaanxi
86	Shaanmai 509	Shaanxi
87	Shaanmai 94	Shaanxi
88	Shaannong 7859	Shaanxi
89	Shaannong 981	Shaanxi
90	Shaanyou 225	Shaanxi
91	Shi 4185	Hebei
92	Shijiazhuang 15	Hebei
93	Shijiazhuang 8	Hebei
94	Shixin 733	Hebei
95	Shixin 828	Hebei
96	Shiyou 17	Hebei
97	Taishan 1	Shandong
98	Taishan 5	Shandong
99	Wan 23094	Anhui
100	Wanmai 19	Anhui
101	Wanmai 29	Anhui
102	Wanmai 33	Anhui
103	Wanmai 38	Anhui
104	Wanmai 50	Anhui
105	Wanmai 52	Anhui
106	Wanmai 53	Anhui
107	Wennong 14	Shandong
108	Wennong 5	Shandong
109	Wunong 148	Shaanxi
110	Xinong 1376	Shaanxi
111	Xinong 2000-7	Shaanxi
112	Xinong 291	Shaanxi
113	Xinong 88	Shaanxi
114	Xinong 9795	Shaanxi
115	Xiaoyan 22	Shaanxi
116	Xiaoyan 54	Shaanxi
117	Xiaoyan 6	Shaanxi
118	Xiaoyan 81	Shaanxi
119	Xinmai 19	Henan

120	Xinmai 940818	Henan
121	Xinmai 9	Henan
122	Su 0663	Anhui
123	Sunong 6	Anhui
124	Yannong 15	Shandong
125	Yannong 18	Shandong
126	Yannong 19	Shandong
127	Yanzhan 4110	Henan
128	Yumai 13	Henan
129	Yumai 18	Henan
130	Yumai 21	Henan
131	Yumai 2	Henan
132	Yumai 34	Henan
133	Yumai 35	Henan
134	Yumai 47	Henan
135	Yumai 49	Henan
136	Yumai 50	Henan
137	Yumai 57	Henan
138	Yumai 63	Henan
139	Yumai 7	Henan
140	Zhengmai 9023	Henan
141	Zhengmai 366	Henan
142	Zhengyin 1	Italy
143	Zhengzhou 3	Henan
144	Zhong 892	Henan
145	Zhongmai 871	Henan
146	Zhongmai 875	Henan
147	Zhongmai 895	Henan
148	Zhongyu 5	Henan
149	Zhongyu 9	Henan
150	Zhou 8425B	Henan
151	Zhoumai 11	Henan
152	Zhoumai 12	Henan
153	Zhoumai 13	Henan
154	Zhoumai 16	Henan
155	Zhoumai 18	Henan
156	Zhoumai 19	Henan
157	Zhoumai 22	Henan
158	Zhoumai 23	Henan
159	Zhoumai 25	Henan
160	Zhoumai 26	Henan

161	Zhoumai 28	Henan
162	Zhoumai 30	Henan
163	Zhoumai 31	Henan
164	Zhoumai 32	Henan
165	Zimai 12	Shandong
166	Zixuan 2	Shandong

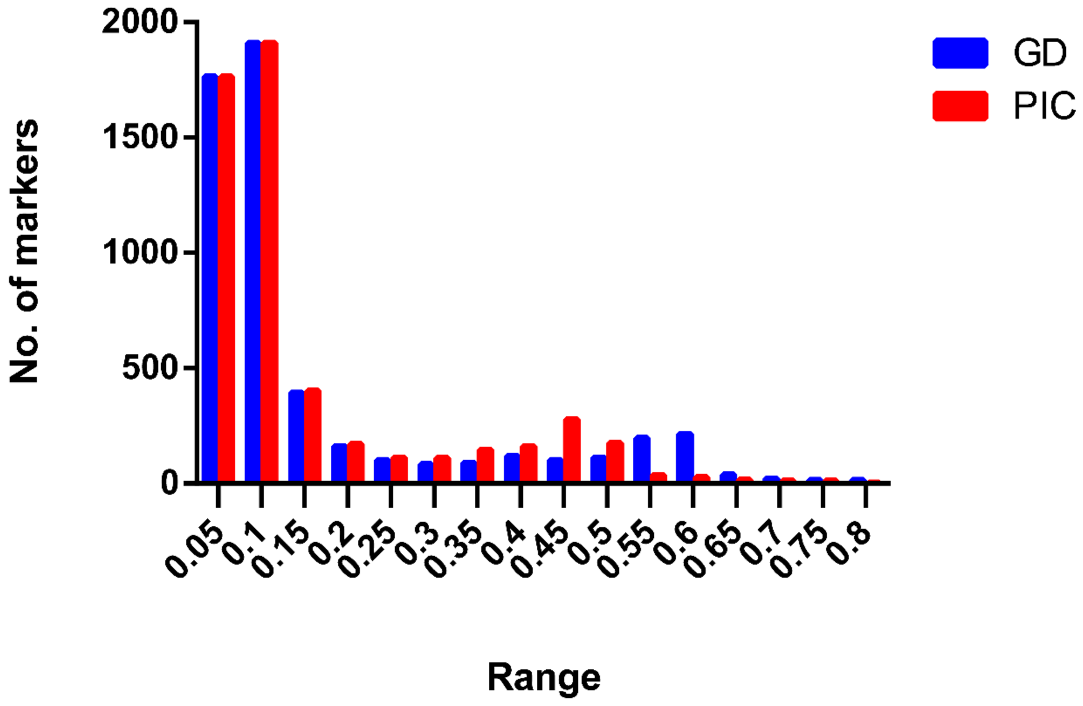

Figure S1. Frequency distribution of gene diversity (GD) and polymorphism information content (PIC) under missing marker rate level of 40%.

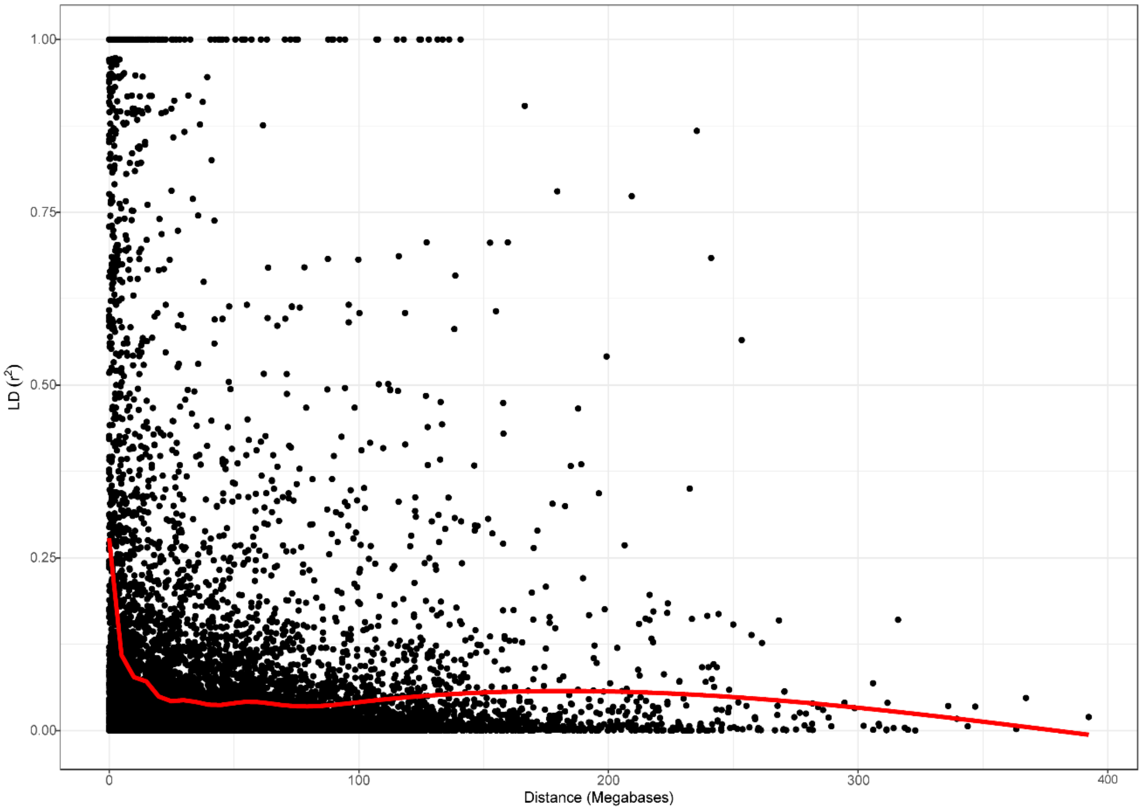

Figure S2. Plot for intra-chromosomal linkage disequilibrium (LD) decay vs genetic distance (Mb) in 166 wheat accessions. LD estimates are reported as squared correlations between markers (r^2).

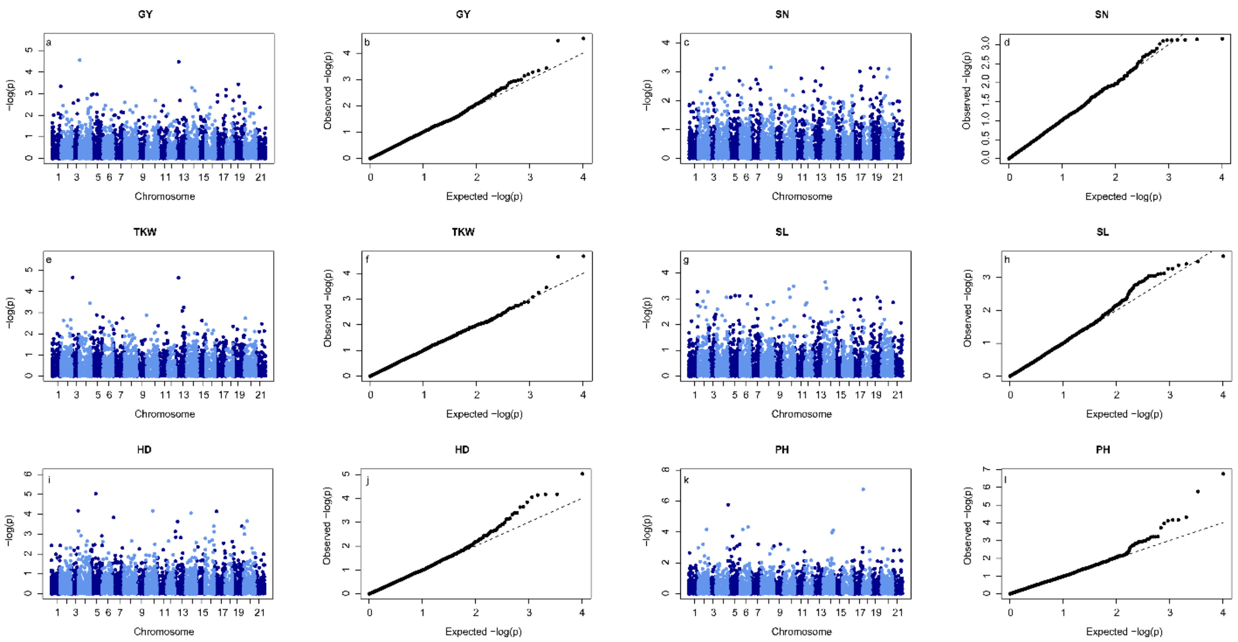

Figure S3. Manhattan (a, c, e, g, i, and k) and quantile-quantile (Q-Q) plots (b, d, f, h, j, and l) from GWAS for yield and yield-related traits under the imputed scenario. GY indicates grain yield; SN, spike number per square meter; TKW, thousand-kernel weight; SL, spike length; HD, heading days; PH, plant height.

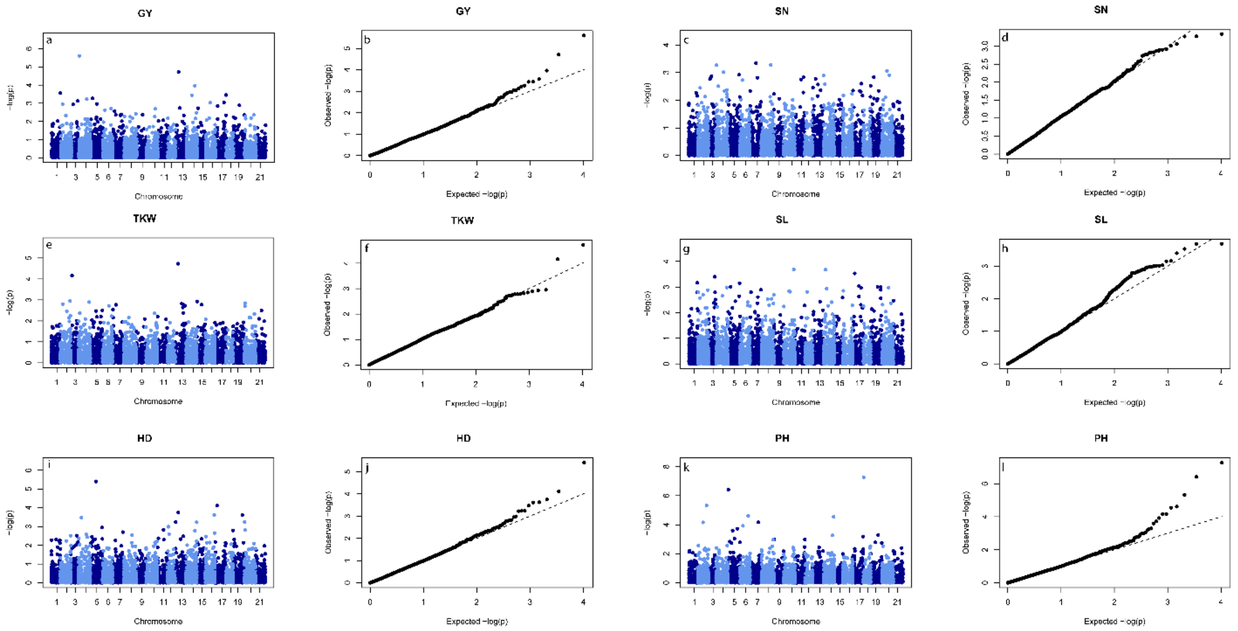

Figure S4. Manhattan (a, c, e, g, i, and k) and quantile-quantile (Q-Q) plots (b, d, f, h, j, and l) from GWAS for yield and yield-related traits under the non-imputed scenario. GY indicates grain yield; SN, spike number per square meter; TKW, thousand-kernel weight; SL, spike length; HD, heading days; PH, plant height.
